# Supplementary material for: ‘You can’t start a car when there’s no petrol left’: a qualitative study of patient, family and clinician perspectives on implantable cardioverter defibrillator deactivation
Source: BMJ Open. 2021 Jul 6;11(7):e048024. doi: 10.1136/bmjopen-2020-048024 (PMC8261879; doi:10.1136/bmjopen-2020-048024)
Supplement: Supplementary data [file bmjopen-2020-048024supp001.pdf]

Appendix: Example interview schedule - prospective deactivation interview

Please note the interview topic guide is a living document. The design is iterative to tailor the interview to the needs of the participant, and the issues that they feel are most important. As analysis occurs concurrently the topic guide is likely to be adapted over time - questions might be added, amended or omitted – and used flexibly.

Plan of interview

1. Introduction
2. Your experience living with an ICD
3. Reasons for deactivating the device
4. Making the decision
5. Improving conversations about deactivation
6. Feedback

1. Introduction

- Thank you agreeing to take part in this interview. As you know, we are interviewing ICD recipients who have made the decision to turn off their device. We are interested in understanding the types of information and support ICD patients need when thinking about device deactivation.
- With your permission I would like to record the interview; all details will be confidential. I am not a clinician so if there are any questions about your device it would be helpful to hear them to understand what you would like more information about but they would be best answered by your doctor or physiologist.
- There are no right or wrong answers and if you feel uncomfortable about any question we can move on to another topic or stop the interview.
- Do you have any questions or concerns?
- *Obtain written consent.*

2. Your experience living with an ICD

- Please can you tell me a little bit about your experience of having an ICD?
- What was your understanding of the device (what does it do/not do)?
- Did your experience a shock from your device at any point?
- How did your ICD impact on your quality of life?)

3. Reasons for deactivating the device

- Can you tell me a little bit about why you wanted your device turned off?
- Were there any other reasons for your decision?

4. Making the decision

- Who did you discuss turning off the device with?
- At what stage did you realise that deactivation was an option?
- What type of information did you receive?
- How was the process of deactivation explained to you?
- Had you ever thought about what would happen with your ICD as you got older? E.g. do you have an advance directive (and was the ICD incorporated into this?)
- What happened after you made the decision to have the device switched off?

- Did your experience match your expectations (how was it different)?

5. Improving conversations about deactivation

- From your experiences what aspects worked well?
- What aspects could have been done better?
- In your view at what stage of the pathway should the possibility of turning the device off be discussed (before/after implantation)?
- Do you have any ideas for how we might improve information giving about device deactivation?
- Who do you think should be involved in these conversations?
- If you could tell patients who are considering getting an ICD anything about what it's like, what would you tell them? What's most important for them to know in making the decision?

6. Feedback

- Thank you for your time.
- What made you take part in this interview?
- Are we asking the right questions?
- Are these the most important issues for you?
- Are there any other issues you would like to discuss to help me to better understand yours and your relative's experiences?
