# Supplementary material for: ‘You can’t start a car when there’s no petrol left’: a qualitative study of patient, family and clinician perspectives on implantable cardioverter defibrillator deactivation
Source: BMJ Open. 2021 Jul 6;11(7):e048024. doi: 10.1136/bmjopen-2020-048024 (PMC8261879; doi:10.1136/bmjopen-2020-048024)
Supplement: Author's manuscript [file bmjopen-2020-048024.draft_revisions.pdf]

BMJ Open

BMJ Open is committed to open peer review. As part of this commitment we make the peer review history of every article we publish publicly available.

When an article is published we post the peer reviewers' comments and the authors' responses online. We also post the versions of the paper that were used during peer review. These are the versions that the peer review comments apply to.

The versions of the paper that follow are the versions that were submitted during the peer review process. They are not the versions of record or the final published versions. They should not be cited or distributed as the published version of this manuscript.

BMJ Open is an open access journal and the full, final, typeset and author-corrected version of record of the manuscript is available on our site with no access controls, subscription charges or pay-per-view fees (http://bmjopen.bmj.com).

If you have any questions on BMJ Open's open peer review process please email info.bmjopen@bmj.com

BMJ Open

'You can't start a car when there's no petrol left': A qualitative study of patient, family, and clinician perspectives on implantable cardioverter defibrillator deactivation

Journal:	*BMJ Open*
Manuscript ID	bmjopen-2020-048024
Article Type:	Original research
Date Submitted by the Author:	16-Dec-2020
Complete List of Authors:	Standing, Holly ; Northumbria University, Faculty of Health and Life Sciences<br>Thomson, Richard; Newcastle University, Population Health Sciences Institute<br>Flynn, Darren; Teesside University, Centre for Rehabilitation, Exercise and Sports Science<br>Hughes, Julian; University of Bristol, Department of Population and Health Sciences<br>Joyce, Kerry; Newcastle University, Population Health Sciences Institute<br>Lobban, Trudie; Arrhythmia Alliance<br>Lord, Stephen; Newcastle Upon Tyne Hospitals NHS Foundation Trust<br>Matlock, Dan D.; University of Colorado, School of Medicine<br>McComb, Janet M; Newcastle Upon Tyne Hospitals NHS Foundation Trust<br>Paes, Paul; Northumbria Healthcare NHS Foundation Trust<br>Wilkinson, Chris; Newcastle University, Population Health Sciences Institute<br>Exley, Catherine; Newcastle University
Keywords:	Pacing & electrophysiology < CARDIOLOGY, Adult palliative care < PALLIATIVE CARE, Heart failure < CARDIOLOGY

SCHOLARONE™
Manuscripts

BMJ

I, the Submitting Author has the right to grant and does grant on behalf of all authors of the Work (as defined in the below author licence), an exclusive licence and/or a non-exclusive licence for contributions from authors who are: i) UK Crown employees; ii) where BMJ has agreed a CC-BY licence shall apply, and/or iii) in accordance with the terms applicable for US Federal Government officers or employees acting as part of their official duties; on a worldwide, perpetual, irrevocable, royalty-free basis to BMJ Publishing Group Ltd ("BMJ") its licensees and where the relevant Journal is co-owned by BMJ to the co-owners of the Journal, to publish the Work in this journal and any other BMJ products and to exploit all rights, as set out in our licence.

The Submitting Author accepts and understands that any supply made under these terms is made by BMJ to the Submitting Author unless you are acting as an employee on behalf of your employer or a postgraduate student of an affiliated institution which is paying any applicable article publishing charge ("APC") for Open Access articles. Where the Submitting Author wishes to make the Work available on an Open Access basis (and intends to pay the relevant APC), the terms of reuse of such Open Access shall be governed by a Creative Commons licence – details of these licences and which Creative Commons licence will apply to this Work are set out in our licence referred to above.

Other than as permitted in any relevant BMJ Author's Self Archiving Policies, I confirm this Work has not been accepted for publication elsewhere, is not being considered for publication elsewhere and does not duplicate material already published. I confirm all authors consent to publication of this Work and authorise the granting of this licence.

For peer review only

'You can't start a car when there's no petrol left': A qualitative study of patient, family, and clinician perspectives on implantable cardioverter defibrillator deactivation

Holly C Standing,[a] Richard G Thomson,[b] Darren Flynn,[c] Julian Hughes,[d] Kerry Joyce,[b] Trudie Lobban,[e] Stephen Lord,[f] Daniel Matlock,[g] Janet M McComb,[f] Paul Paes,[h] Chris Wilkinson,[b] Catherine Exley[b]

Author affiliations:

[a] Department of Nursing, Midwifery and Health, Faculty of Health and Life Sciences, Northumbria University, Newcastle upon Tyne, UK

[b] Population Health Sciences Institute, Faculty of Medical Sciences, Newcastle University, Newcastle upon Tyne, UK

[c] Centre for Rehabilitation, Exercise and Sports Science, School of Health and Life Sciences, Teesside University, Tees Valley, UK

[d] Department of Population and Health Sciences, Bristol Medical School, University of Bristol, Bristol, UK

[e] Arrhythmia Alliance, Chipping Norton, Oxfordshire, UK

[f] Newcastle upon Tyne Hospitals NHS Foundation Trust, Newcastle upon Tyne, UK

[g] Department of Medicine, University of Colorado School of Medicine, Aurora, CO, USA

[h] Northumbria Healthcare NHS Foundation Trust, North Shields, UK

Corresponding author:

Professor Catherine Exley, Dean of Population Health Sciences Institute

Email: catherine.exley@ncl.ac.uk

Telephone: +44 (0) 191 2088851

Population Health Sciences Institute, Faculty of Medical Sciences, Newcastle University, Newcastle upon Tyne, NE1 7RU

For peer review only

Abstract

Objective: To explore the attitudes towards implantable cardioverter defibrillator (ICD) deactivation and initiation of deactivation discussions amongst patients, relatives and clinicians.

Design: A multi-phase qualitative study consisting of in-situ hospital ICD clinic observations, and semi-structured interviews of clinicians, patients, and relatives. Data were analysed using a constant comparative approach.

Setting: One tertiary and two district general hospitals in England.

Participants: We completed 38 observations of hospital consultations prior to ICD implantation, and 80 interviews with patients, family members, and clinicians between 2013 and 2015. Patients were recruited from pre-implantation to post-deactivation. Clinicians included cardiologists, cardiac physiologists, heart failure nurses, and palliative care professionals.

Results: Four key themes were identified from the data: the current status of deactivation discussions; patients' perceptions of deactivation; who should take responsibility for deactivation discussions and decisions; and timing of deactivation discussions. We found that although patients and doctors recognised the importance of advance care planning, including ICD deactivation at an early stage in the patient journey, this was often not reflected in practice. The most appropriate clinician to take the lead was thought to be dependent on the context, but could include any appropriately trained member of the healthcare team. It was suggested that deactivation should be raised pre-implantation and regularly reviewed. Identification of trigger points post-implantation for deactivation discussions may help ensure that these are timely and inappropriate shocks are avoided.

Conclusions: There is a need for early, ongoing, and evolving discussion between ICD recipients and clinicians regarding the eventual need for ICD deactivation. The most appropriate clinician to instigate deactivation discussions is likely to vary between patients and models of care. Reminders at key trigger points, and routine discussion of deactivation at implantation and during advance care planning could prevent distressing experiences for both the patient and their family at the end-of-life.

For peer review only - http://bmjopen.bmj.com/site/about/guidelines.xhtml

Strengths and limitations of this study (up to five)

- Implantable cardioverter defibrillator implants are increasing and existing recipients are ageing, which makes optimal end-of-life planning an important and topical issue.
- This is the first study of its kind to include both clinic observations and semi-structured interviews.
- There was a robust recruitment strategy, with a large number of participants enrolled.
- However, this study lacks the perspectives of primary care clinicians.
- A limited number of patients that were actively considering deactivation was recruited.

For peer review only

Introduction

Implantable cardioverter defibrillators (ICDs) improve survival in patients at high risk of sudden cardiac death through delivering treatments to terminate life-threatening arrhythmia.[1-3] However, as people age the risk of non-arrhythmic modes of death, such as frailty, progressive heart failure, or other terminal diseases such as cancer, may dominate, and the potential benefit from an ICD is reduced.[4-6] More than one third of hospitalised patients with an ICD in situ experience a ventricular tachyarrhythmia within the last hour of life,[7] and therefore leaving the device active is likely to lead to shocks at the end-of-life, which may be distressing, painful, and futile.[8-12] Advance care planning (ACP) aims to ensure that appropriate measures are in place to aid patients' transition to end-of-life care.[13] Although guidelines suggest that ongoing ICD activation should be considered as part of a patient's care goals,[14 15] clinicians may be failing to engage patients sufficiently in ACP.[9 16]

A proactive approach recommended by international guidelines mandates deactivation discussion prior to implantation as a part of shared decision making,[14 17 18] This has the declared support of both clinicians and patients.[10 12 19] Yet there is evidence that this discussion does not routinely occur.[20 21] Failure to engage patients may ultimately put undue pressure on family members to make deactivation decisions; more than half of deactivation requests come from family members.[22] Previous work has identified that patients recall the positive, 'life-saving' attributes of ICD discussions, and that pre-implant end-of-life ACP discussions were limited.[23 24] In this study, we add to the existing evidence base by including non-participant observations in addition to semi-structured interviews. The study provides a comprehensive exploration of patient, family member and clinician attitudes towards deactivation discussions. In addition, we explored when and how best to approach and undertake such discussions.

For peer review only - http://bmjopen.bmj.com/site/about/guidelines.xhtml

Methods

Study design

The study comprised two phases: clinical observations, and interviews with patients, family members, and clinicians. Ethical approval was granted by NHS Research Ethics Committee North East-Sunderland (Reference: 13/NE/0105). All participants provided written informed consent. The investigation conforms with the principles outlined in the Declaration of Helsinki.[25] Additional details of the methods, including further description and justification of study sites, are provided in the full report.[26]

Patient and Public Involvement

Public and patient engagement was initiated prior to the development of the outline application, and their feedback guided the research question and study approach. The Founder and Trustee of Arrhythmia Alliance (TL) was a co-applicant and member of our study advisory group, and provided service user input into all stages, including co-authoring this manuscript and the report to the study funders. Two patient/carer representatives (with an interest in end-of-life issues) joined the initial project advisory group. The Arrhythmia Alliance and the North of England Cardiovascular Network reviewed the research proposal, and their comments were incorporated.

Sampling and recruitment

Phase 1: Observations

Non-participant observations [27] were conducted in clinics across three hospitals: one tertiary care (implanting) centre, and two district general hospitals. Observations at the tertiary care centre were with two implanting cardiologists, and observations at district general hospitals were with two heart failure nurses and one non-implanting cardiologist. Observations allowed

For peer review only - http://bmjopen.bmj.com/site/about/guidelines.xhtml

researchers (HS/KJ) to familiarise themselves with the clinical environment and decision-making about ICDs. Field notes informed the sampling strategy and interview schedules for phase two.

Cardiologists identified patients attending to discuss ICD implantation. Opt-out consent was used for this phase; study information was mailed with appointment letters, explaining that a researcher might be present to observe their consultation. An opt-out slip was included, and patients were asked to present this upon arrival if they chose not to participate.

Phase 2: Interviews

Clinician interviews

Clinicians were recruited from five hospitals in the north of England with interviews, conducted over the telephone or at the clinician's place of work, exploring: the referral pathway for ICD implantation; approaches to risk communication; current approaches to, and people involved in, decision-making; and timing of deactivation discussions. Purposive sampling[28] was used to capture a range of perspectives from specialities involved in the care of ICD patients.

Patient and family member interviews

We sought to include a range of patient experiences from those considering ICD implantation to those recently deactivated, as well as bereaved family members of ICD recipients 4-18 months post bereavement. The interview schedule covered issues identified through the observations and a literature review (see supplementary material), including understandings of, and feelings about, the ICD; decision-making about implantation; whether, how, and by whom deactivation had been discussed; and preferences for information and decision support.

For peer review only - http://bmjopen.bmj.com/site/about/guidelines.xhtml

For peer review only

Next-of-kin contact details for deceased patients with an ICD were identified and an information pack was mailed, including an introductory letter from the implanting cardiologist. The letter invited interested individuals to return a consent-to-contact form to the research team. When received, the researcher arranged an interview. Invitations offered the option of being interviewed with a friend or family member, or passing the invitation to someone more closely involved in the patient's care. Interviews were conducted at the participants' home or over the telephone and were audio-recorded, transcribed verbatim and anonymised.

Data analysis

Data collection was guided by the constant comparative method.[29] Data collection and analysis ran concurrently throughout the study. Analysis of early interviews informed the interview schedule for subsequent interviews, and analysis of early transcripts were revised as data collection progressed. We critically examined the different perspectives and experiences of those involved in decision making about deactivation of ICDs. NVivo version 8 (QSR International, Warrington UK) was used to facilitate data management. Data was analysed by three experienced qualitative researchers (HS and KJ, with support from CE). Regular meetings were held with the rest of the research team and the advisory group to discuss themes derived from the data. All names are pseudonyms.

For peer review only - http://bmjopen.bmj.com/site/about/guidelines.xhtml

Findings

Between July 2013 and January 2015, 38 observations of consultations led by heart failure nurses, non-implanting and implanting cardiologists were conducted. Consultations lasted 10 to 30 minutes.

Forty-four patient interviews were conducted in 33 men and 11 women, aged between 47 and 85 years. Most patients (n=34) had been offered an ICD for primary prevention (patients without a history of sustained ventricular tachycardia or sudden cardiac arrest),[18] the remainder (n=10) were for secondary prevention (with a history of sustained tachycardia or sudden cardiac arrest).[18] Table 1 provides a breakdown of patient, bereaved relative (n=7) and clinicians (n=29).

Patient and bereaved relative interviews ranged from 11-113 (mean 44) minutes, clinician interviews from 20-83 (mean 42) minutes.

For peer review only - http://bmjopen.bmj.com/site/about/guidelines.xhtml

Table 1: Participant characteristics (interviews)

**Patients**	**n**
Pre-implantation	13
Declined ICD	8
Post-implantation	21
Deactivated	2
**Total**	**44**
**Bereaved relatives**	
Bereaved spouse	4
Bereaved spouse and daughter	2
Bereaved son and daughter-in-law	1
**Total**	**7**
**Clinicians**	
Implanting cardiologist	5
Non-implanting cardiologist	5
Arrhythmia nurse	1
Heart failure nurses (hospital and community)	6
Cardiac physiologists*	4
Health psychologists	2
Palliative care specialists	6
**Total**	**29**
**Overall total**	**80**

* A cardiac physiologist is a clinical scientist, known as a device (ICD or pacemaker) technician in the US.

For peer review only

Four key themes were identified: the current status of deactivation discussions; patients' perceptions of deactivation; responsibility for deactivation discussions and decisions; and timing of deactivation discussions.

Current status of deactivation discussions

Clinicians recognised the importance of timely ICD deactivation to avoid patients experiencing unnecessary shocks towards the end-of-life; however current management of ICD deactivation was considered to be sub-optimal at times. Patients were not consistently engaged in deactivation discussions and when this did occur, the patient was often close to death. Failure to deactivate the ICD in a timely manner could result in stressful and upsetting situations.

> *I think the most recent one that we've had on the ward […] the decision was left too late, the patient was getting shocked too frequently, and nobody had had the chat to the family to say, "Well this is the defibrillator doing all of this, distressing them, and what we can do about it".* (Dr J, non-implanting cardiologist)

> *the patient in my view at that stage was actually dead, or was on the verge of being dead and it* [ICD] *was still delivering… Then once it had been deactivated, the patient passed away in a matter of a few moments. It wasn't pleasant.* (Nurse C, heart failure nurse)

For peer review only - http://bmjopen.bmj.com/site/about/guidelines.xhtml

Bereaved relatives felt that deactivation was often poorly handled. One suggested reason for this was clinicians' apparent reticence to deactivate the ICD prior to the patient being in the last hours of life.

> *she [cardiologist] said his heart looked alright and she didn't feel it was time to deactivate it … She [cardiologist] said to me "Do you think that he's ready to be deactivated?" And at the time I suppose I didn't, I said no …So that wasn't a very nice experience.* (Shirley Bereaved wife)

Despite having engaged in ACP with a heart failure nurse, the patient's preference for deactivation was questioned by the implanting cardiologist who deferred the decision to the patient's wife. Although the ICD was eventually deactivated, it was reported that this occurred only hours before death, following intervention by the heart failure nurse. The patient's daughter, who also participated in the interview, felt it had been inappropriate for the cardiologist to put her mother in a position where she was expected to make an immediate deactivation decision.

Some patients reported facing apparent resistance from clinicians when trying to express their preference for deactivation.

> *What he [the cardiologist] actually said [was], 'well, you'll basically drop down dead as soon as I switch it off'. It was so unkind. I wouldn't dare say that to anyone.* (Fred, ICD deactivated)

For peer review only - http://bmjopen.bmj.com/site/about/guidelines.xhtml

This reported response from the cardiologist caused the patient and his wife a great deal of distress, and misrepresented the function of the ICD. The cardiologist's actions were perceived as an attempt to mislead the patient regarding the outcome of deactivation, so he would reconsider his decision to decline further medical intervention. This patient had declined his cardiologist's recommendation to be placed on the waiting list for heart transplant owing to previous experiences with organ transplantation and the associated side effects; the patient accepted he was approaching end-of-life and wanted limited intervention. Rather than exploring the patient's reasons for requesting deactivation, this account would seem to demonstrate a failure to undertake SDM.

Patients' perceptions of deactivation

Understanding of, and feelings about, deactivation amongst patients and family members were variable. Some appeared very pragmatic:

> *You can't fire, start a car when there's no petrol in it, yeah? … it's no good firing your starter motor, starting your car, when there's no petrol in it.* (Janet, bereaved relative)

For these individuals, deactivation was not necessarily viewed negatively, it was a means of avoiding unpleasant and unnecessary shocks:

> *There are the end-of-life considerations* [..] *I'm aware that, that this could happen* [deactivation]. *I think, yes, if I was seriously ill and near the end-of-life. I wouldn't want the thing shocking me*. (Isobel, post-implantation)

The knowledge that deactivation may be a possibility could even offer a sense of control. However, for some patients the idea of deactivation was of concern. The device could become a source of comfort, which patients wished to keep activated as long as possible. This was particularly apparent amongst those who had recently had their ICD implanted.

> Burt: *If you were to take it off us now I'd miss it*
>
> Interviewer: *So* [you] *would never think of switching it off?*
>
> Burt: *No, no, no* (Burt, post-implantation)

Appropriate timing of deactivation discussions

Pre-implantation

Pre-implantation offers the first opportunity to introduce deactivation. It was recognised that discussing deactivation could influence a patient's decision on implantation. A few patients indicated that, had they been aware of the ICD's potential to influence their mode of death and the possible need for future deactivation, this may have affected their decision making.

> *if it had been mentioned, I might have well have thought about not having one* (Adrian, post-implant)

Although most patients expressed a preference to be informed about deactivation prior to implantation, some clinicians were less convinced. They felt that discussions about deactivation and end-of-life, even when hypothetical, conflicted with perceived patient needs and expectations of doctor-patient encounters.

For peer review only - http://bmjopen.bmj.com/site/about/guidelines.xhtml

I mean most patients don't want to talk about it [EOL]… they come to see you 'cause they want to get better. They don't want to be told, "Well, you're going to die." (Dr B, non-implanting cardiologist)

Introducing deactivation pre-implantation was viewed as an 'illogical juxtaposition', which might give mixed messages and cause distress. ICDs are offered as a potentially lifesaving intervention; to discuss end-of-life was seen as inappropriate and potentially confusing. Clinicians also expressed concerns that patients might develop misconceptions that deactivation would result in immediate death.

I suppose also this is something to do with your heart and how people comprehend that, so it seems it can be a lot more immediate and a lot more life-threatening even at the end-of-life. (Dr D, palliative care clinician)

Questions were also raised about patients' ability to remember information about deactivation given pre-implantation. Pre-implantation is often a time of high stress, when patients are expected to digest a considerable amount of information. Expecting patients to absorb and process information about deactivation may be unrealistic.

But I don't think that people at that point [implantation] *can really take on board that at some distant point in the future that they might need it changing* (Dr E, palliative care clinician)

For peer review only - http://bmjopen.bmj.com/site/about/guidelines.xhtml

Ongoing and evolving discussions

Rather than one-off conversations, both patients and clinicians indicated that deactivation should be an ongoing and evolving discussion, where the appropriateness of the ICD and how it fits with the patient's life is regularly reassessed. It was thought that 'sowing the seed' of deactivation pre-implantation might facilitate later conversations.

> *you have to sow the seed, you know, plant the seed about what might happen in the future.* (Nurse C, heart failure nurse)

The patient's need, and referral for, an ICD could be an initial prompt to introduce the concept of deactivation, which could then be revisited at intervals. One participant suggested that 'key points in admissions' could be used as triggers for deactivation discussions. Hospital and hospice admissions, and ICD firing or replacement, could be prompts for deactivation discussions. Inclusion of the patient on palliative care registers and production of ACP documentation, such as a do not attempt cardiopulmonary resuscitation form, could be used as final trigger points where deactivation must be discussed. This approach could avoid patients 'falling through the gaps', ensuring that every patient is engaged in deactivation discussions in a timely and appropriate manner.

Responsibility for deactivation discussions and decisions

Heart failure nurses, implanting cardiologists, physiologists, palliative care clinicians, and primary care clinicians may all have a role in both advance deactivation discussions and the ultimate decision to deactivate the ICD. However, no single clinical group currently took primary responsibility for these tasks.

For peer review only - http://bmjopen.bmj.com/site/about/guidelines.xhtml

Although cardiologists possess a high level of expertise that may facilitate them engaging patients in these discussions, their role is traditionally focused on saving patients.

> *I think doctors don't like talking about dying, because it's a sign, it's a sign that you have failed* (Dr F, implanting cardiologist)

Further, depending on the patient's condition, contact with the cardiologist following implantation of the device may be infrequent, and so their capacity to have an ongoing role in deactivation discussions may be limited.

Cardiac physiologists have regular contact with ICD patients, offering an opportunity to engage patients in ongoing discussions. However, some of the physiologists interviewed appeared reticent to engage in this conversation.

> *So although I think we are in a good position in some ways, I think maybe there are other professionals out there who are in a position to do it as well.* (Mr G, cardiac physiologist)

This reticence may be related to a feeling that they lacked the requisite skills to engage patients in SDM about continuation of ICD therapy. Cardiac physiologists' confidence and comfort with regards to these responsibilities may be improved by additional training.

> *it's such a hard thing to do* [discussing deactivation and death]. *Probably need some training on how to broach the subject and when to broach it, and what to say.* (Ms I, cardiac physiologist)

For peer review only - http://bmjopen.bmj.com/site/about/guidelines.xhtml

Heart failure nurses felt they were already undertaking the work of engaging patients in deactivation discussions.

> *I have quite a few interesting conversations about death when I'm talking about defibrillators … it's just a simple sentence, 'your device will be switched off at some point, you can't live forever'* (Nurse H, heart failure nurse)

Clinicians recognised that there are various professionals who could have a role in engaging patients in deactivation discussions.

> *I think for any given patient somebody's going to be taking the lead, and it's not always clear-cut by role who that should be, so it may either be the GP* [primary care physician]*, it's conceivably a heart failure nurse, it's conceivably a MacMillan* [palliative care] *nurse or our palliative care service* (Dr M, palliative care clinician)

For peer review only - http://bmjopen.bmj.com/site/about/guidelines.xhtml

Discussion

Our findings demonstrate that clinicians understand the importance of deactivation discussions with patients and their families, and ACP for people with ICDs. Both patients and clinicians recognised the need for improvement; discussions about deactivation are often occurring late or not at all, resulting in negative impacts on patients and their families. We outline approaches for ensuring that these important and sensitive conversations take place ahead of time, based upon extensive observations and semi-structured interviews.

It has previously been shown in survey evidence from the USA,[30] and qualitative data from Sweden,[23] and Northern Ireland[24 31] that patients report limited knowledge about deactivation, which is rarely discussed pre-implantation. Yet, SDM should involve bidirectional knowledge transfer between the patient and their families, and clinicians, including elicitation of individual patient preferences and values for different outcomes.[32] Indeed, informed consent requires that patients understand the risks, benefits and consequences of interventions in the light of what is important to them in making such decisions.[33] Our findings indicate a mismatch between patients' desire to be informed about deactivation and clinicians' perception of patients' information preferences, particularly pre-implantation. Many of our patient interviewees indicated they would be comfortable discussing deactivation and believed they should be informed about this before implantation so they had appropriate expectations of the future. Knowing about the possibility of future deactivation could afford patients a sense of control. However, whilst some clinicians perceived that patients did not want early deactivation discussion, and did not routinely pursue it during consultations, our results suggest that many patients do. Furthermore, the absence of such discussion may result in patient decisional regret and contribute to late

For peer review only - http://bmjopen.bmj.com/site/about/guidelines.xhtml

decisions about deactivation. Another potential explanation is that clinicians feel uncomfortable discussing death in a consultation that is about preventing death. Knowing that many patients wish to be engaged in this discussion may help clinicians explore this pre-implantation with more confidence.

Previous research has also demonstrated greater discomfort amongst clinicians about ICD deactivation compared to the withdrawal of other life sustaining therapies[34], and ICDs are not considered in the same context as other end-of-life decisions.[11] It is possible that ethical questions about deactivation of the ICD as a form of euthanasia may contribute to some of the unease around initiating the relevant discussions.[9 35] Clinicians' reluctance to engage in end-of-life discussions may also stem from fear and anxiety of accepting the limits of their ability to save patients.[36]

Our findings suggest a need for ongoing and evolving deactivation discussions, where the issue is introduced pre-implantation (as desired by the majority of patients) and built upon through subsequent encounters. Regularly revisiting ACP in relation to cardiac devices will enable clinicians to meet patients' goals of care better.[37] Prompts at appropriate points may help clinicians and patients to engage in these discussions.[38] Interventions which include education, clinical tools and standardised electronic health record templates have increased rates of ICD deactivation and clinicians' confidence in managing the device.[39] The possibility of empowering patients and families to initiate the discussion themselves should also be considered.

Our findings demonstrate a lack of consensus amongst clinicians regarding responsibility for deactivation discussions. Heart failure nurses often have established relationships and regular

For peer review only - http://bmjopen.bmj.com/site/about/guidelines.xhtml

contact with patients and their families, which may facilitate them in aligning discussions with the patient's values and preferences, and building upon the discussion over time. As others, [9 40] we suggest that a one-size-fits-all approach is not necessarily appropriate; the clinician most appropriate to discuss deactivation may differ between patients and at different times. Different models of care delivery for ICD patients will also have an influence; where specialist arrhythmia nurses are available, they will have an important role in deactivation discussions. However, these services were unavailable to the majority of our participants, resulting in more demand on the cardiologists and cardiac physiologists. Our findings support other research, which has suggested an inter-professional approach to initiating deactivation discussions.[41] Whoever is involved in care must attempt to ensure that patients do not fall through the gaps if clinicians fail to engage patients proactively in these discussions; and whichever clinicians are involved, they should have sufficient training, skills and confidence to undertake the task. Development of clear guidelines outlining the responsibility of each clinician group would provide clarity about expectations to engage in this work and ensure timely discussions. Additionally, we recognise that specialist-led secondary care clinics may lack access to comprehensive clinical information required for a holistic ongoing assessment of an individual and their circumstances. As such, we recognise the important role that general practitioners have in ACP, and the need for further data on how to optimise the interface between primary and secondary care with regard to ICD care.

Strengths and limitations

This article is from a large qualitative study combining observations and interviews with a range of stakeholders. The observations allowed familiarity with the clinical setting; this also allowed us to refine our sampling strategies and interview schedule for the second phase of the study (interviews). The range of participants sampled provides insight into the

For peer review only - http://bmjopen.bmj.com/site/about/guidelines.xhtml

perspectives of various clinician groups, as well as those of patients and their family members. However, we recognise the limitations of our work. Few interviews were conducted with patients with a deactivated ICD, or considering deactivation, because there is often a short time between deactivation and death.[31] Significant attempts were made to observe clinic appointments where decisions about ICD deactivation were made, and engage these patients in interviews, but success in recruiting this group was limited. Recruitment of bereaved relatives also proved challenging. The inclusion period for this group was expanded from 4-6 months following bereavement to 4-18 months, which improved our response rate. However, some of these participants may have been less able to recall events surrounding deactivation of the ICD (or not) and death, given the length of time post-bereavement. Finally, this study did not include primary care settings; future research should explore the views and experiences of primary care clinicians regarding their role in discussions and decision-making around ICD deactivation.

For peer review only

For peer review only - http://bmjopen.bmj.com/site/about/guidelines.xhtml

Conclusions

Our study has identified significant unmet need, particularly in the area of deactivation discussion. We believe that patients and clinicians need more support to do this well. We believe that further work is needed to identify the best way of doing it, which may include further education, protocols/guidelines and reminders (for both clinicians and patients) to have such discussions at key points.

For peer review only

For peer review only - http://bmjopen.bmj.com/site/about/guidelines.xhtml

Acknowledgements

We would like to thank all the patients, relatives, and clinicians who allowed us to observe their consultations and took part in the interviews and workshops. We are grateful to the members of the project advisory group for their valuable advice throughout the study.

Funding

This work was supported by The National Institute for Health Research Health Services and Delivery Research programme.

Competing interests

None declared.

Contributions

Developing the study protocol: CE, JH, KJ, TL, SL, DM, JM, RT; Patient and public involvement: KJ; Developing the study design: CE, JH, KJ, TL, SL, DM, PP, RT; Data collection: HS, DF, KJ; Data interpretation: HS, CE, DF, JH, TL, SL, DM, PP, CW, RT. All authors were involved in drafting the work or revising it critically for important intellectual content. All authors approved the final version.

For peer review only

References

1. Bardy GH, Lee KL, Mark DB, et al. Amiodarone or an implantable cardioverter-defibrillator for congestive heart failure. *N Engl J Med* 2005;352(3):225-37. doi: 10.1056/NEJMoa043399 [published Online First: 2005/01/22]
2. Kadish A, Dyer A, Daubert JP, et al. Prophylactic defibrillator implantation in patients with nonischemic dilated cardiomyopathy. *N Engl J Med* 2004;350(21):2151-8. doi: 10.1056/NEJMoa033088 [published Online First: 2004/05/21]
3. Moss AJ, Zareba W, Hall WJ, et al. Prophylactic implantation of a defibrillator in patients with myocardial infarction and reduced ejection fraction. *N Engl J Med* 2002;346(12):877-83. doi: 10.1056/NEJMoa013474 [published Online First: 2002/03/22]
4. Schleifer JW, Shen W-K. Implantable Cardioverter-Defibrillator Implantation, Continuation, and Deactivation in Elderly Patients. *Current Geriatrics Reports* 2017;6(4):279-89. doi: 10.1007/s13670-017-0226-9
5. Goonewardene M, Barra S, Heck P, et al. Cardioverter-defibrillator implantation and generator replacement in the octogenarian. *EP Europace* 2014;17(3):409-16. doi: 10.1093/europace/euu248
6. Walker DM, Gale CP, Lip G, et al. Editor's Choice - Frailty and the management of patients with acute cardiovascular disease: A position paper from the Acute Cardiovascular Care Association. *Eur Heart J Acute Cardiovasc Care* 2018;7(2):176-93. doi: 10.1177/2048872618758931 [published Online First: 2018/02/17]
7. Kinch Westerdahl A, Sjoblom J, Mattiasson AC, et al. Implantable cardioverter-defibrillator therapy before death: high risk for painful shocks at end of life. *Circulation* 2014;129(4):422-9. doi: 10.1161/circulationaha.113.002648 [published Online First: 2013/11/19]
8. Garcia M, Wanagat J. Cardiovascular Implantable Electronic Devices in End of Life Care. *Proceedings of UCLA Healthcare* 2014;18
9. Stoevelaar R, Brinkman-Stoppelenburg A, van Driel AG, et al. Implantable cardioverter defibrillator deactivation and advance care planning: a focus group study. *Heart* 2020;106(3):190-95. doi: 10.1136/heartjnl-2019-315721
10. Westerdahl AK, Sutton R, Frykman V. Defibrillator patients should not be denied a peaceful death. *International journal of cardiology* 2015;182:440-46.
11. Goldstein N, Mehta D, Teitelbaum E, et al. “It’s Like Crossing a Bridge” Complexities Preventing Physicians from Discussing Deactivation of Implantable Defibrillators at the End of Life. *J GEN INTERN MED* 2008;23(1):2-6. doi: 10.1007/s11606-007-0237-x
12. Kelley AS, Mehta SS, Carrington Reid M. Management of Patients with ICDs at the End of Life (EOL): A Qualitative Study. *American Journal of Hospice and Palliative Medicine* 2009;25(6):440-46. doi: 10.1177/1049909108320885
13. National End of Life Care Programme. Capacity, Care Planning and Advance Care Planning in Life Limiting Illness a Guide for Health and Social Care Staff. Leicester: Department of Health, Natational End of Life Care Programme, 2014.
14. Pitcher D, Soar J, Hogg K, et al. Cardiovascular implanted electronic devices in people towards the end of life, during cardiopulmonary resuscitation and after death: guidance from the Resuscitation Council (UK), British Cardiovascular Society and National Council for Palliative Care. *Heart* 2016;102(Suppl 7):A1-A17. doi: 10.1136/heartjnl-2016-309721
15. Beattie J. ICD deactivation at the end of life: principles and practice. A dicussion document for healthcare professionals: British Heart Foundation, 2013.
16. Norekvål TM, Kirchhof P, Fitzsimons D. Patient-centred care of patients with ventricular arrhythmias and risk of sudden cardiac death: What do the 2015 European Society of Cardiology guidelines add? *European Journal of Cardiovascular Nursing* 2017;16(7):558-64. doi: 10.1177/1474515117702558
17. Padeletti L, Arnar DO, Boncinelli L, et al. EHRA Expert Consensus Statement on the management of cardiovascular implantable electronic devices in patients nearing end of life or requesting withdrawal of therapy. *Europace : European pacing, arrhythmias, and cardiac electrophysiology : journal of the working groups on cardiac pacing, arrhythmias, and*

For peer review only - http://bmjopen.bmj.com/site/about/guidelines.xhtml

cardiac cellular electrophysiology of the European Society of Cardiology 2010;12(10):1480-9. doi: 10.1093/europace/euq275 [published Online First: 2010/08/03]
18. Al-Khatib SM SW, Ackerman MJ, Bryant WJ, Callans DJ, Curtis AB, Deal BJ, Dickfeld T, Field ME, Fonarow GC, Gillis AM, Hlatky MA, Granger CB, Hammill SC, Joglar JA, Kay GN, Matlock DD, Myerburg RJ, Page RL,. 2017 AHA/ACC/HRS Guideline for Management of Patients With Ventricular Arrhythmias and the Prevention of Sudden Cardiac Death,. *Circulation* 2017
19. Raphael CE, KOA-WING M, Stain N, et al. Implantable Cardioverter-Defibrillator Recipient Attitudes towards Device Deactivation: How Much do Patients Want to Know? *Pacing and clinical electrophysiology* 2011;34(12):1628-33.
20. Marinskis G, van Erven L. Deactivation of implanted cardioverter-defibrillators at the end of life: results of the EHRA survey. *Europace : European pacing, arrhythmias, and cardiac electrophysiology : journal of the working groups on cardiac pacing, arrhythmias, and cardiac cellular electrophysiology of the European Society of Cardiology* 2010;12(8):1176-7. doi: 10.1093/europace/euq272 [published Online First: 2010/07/29]
21. Hill L, McIlfatrick S, Taylor B, et al. P2983Who decides what and when: a holistic understanding of decision-making in implantable cardioverter defibrillator deactivation. *European Heart Journal* 2017;38(suppl_1)
22. Buchhalter LC, Ottenberg AL, Webster TL, et al. Features and outcomes of patients who underwent cardiac device deactivation. *JAMA Internal Medicine* 2014;174(1):80-85. doi: 10.1001/jamainternmed.2013.11564
23. Ågård A, Löfmark R, Edvardsson N, et al. Views of patients with heart failure about their role in the decision to start implantable cardioverter–defibrillator treatment: prescription rather than participation. *Journal of Medical Ethics* 2007;33(9):514-18. doi: 10.1136/jme.2006.017723
24. Hill LM, McIlfatrick S, Taylor B, et al. Implantable cardioverter defibrillator (ICD) functionality: patient and family information for advanced decision-making. *BMJ Supportive & Palliative Care* 2019:bmjspcare-2019-001835. doi: 10.1136/bmjspcare-2019-001835
25. World Medical Association. Declaration of Helsinki: ethical principles for medical research involving human subjects. *Jama* 2013;310(20):2191-4. doi: 10.1001/jama.2013.281053 [published Online First: 2013/10/22]
26. Standing H, Exley C, Flynn D, et al. A qualitative study of decision-making about the implantation of cardioverter defibrillators and deactivation during end-of-life care. *Health Services and Delivery Research* 2016;4(32)
27. Hammersley M, Atkinson P. Ethnography: Principles in practice: Routledge 2007.
28. Patton MQ. Qualitative Evaluation and Research Methods. Thousand Oaks: SAGE Publications 1990.
29. Charmaz K. Constructing Grounded Theory: A Practical Guide Through Qualitative Analysis. Thousand Oaks: Sage Publications 2006.
30. McEvedy SM, Cameron J, Lugg E, et al. Implantable cardioverter defibrillator knowledge and end-of-life device deactivation: A cross-sectional survey. *Palliative medicine* 2018;32(1):156-63. doi: 10.1177/0269216317718438 [published Online First: 2017/07/06]
31. Hill L, McIlfatrick S, Taylor B, et al. Patients' perception of implantable cardioverter defibrillator deactivation at the end of life. *Palliative medicine* 2015;29(4):310-23. doi: 10.1177/0269216314550374 [published Online First: 2014/09/23]
32. Spatz ES, Spertus JA. Shared decision making: a path toward improved patient-centered outcomes. *Circulation Cardiovascular quality and outcomes* 2012;5(6):e75-7. doi: 10.1161/circoutcomes.112.969717 [published Online First: 2012/11/22]
33. Court TS. Montgomery (Appellant) v Lanarkshire Health Board (Respondent) (Scotland). In: Court TS, ed. UKSC 11. https://www.supremecourt.uk/cases/uksc-2013-0136.html, 2015.
34. Kramer DB, Kesselheim AS, Brock DW, et al. Ethical and legal views of physicians regarding deactivation of cardiac implantable electrical devices: a quantitative assessment. *Heart rhythm* 2010;7(11):1537-42. doi: 10.1016/j.hrthm.2010.07.018 [published Online First: 2010/07/24]
35. Lebner A. Deactivating implantable cardioverter-defibrillators (ICDs) in cancer patients: A scoping review of ethical considerations. *Journal of Pain Management* 2017;10(1):99.

For peer review only - http://bmjopen.bmj.com/site/about/guidelines.xhtml

36. MacKenzie MA. Magical thinking around end of life: An editorial on ‘Implantable Cardioverter Defibrillator deactivation discussions: Reality vs. recommendations’. *European Journal of Cardiovascular Nursing* 2016;15(1):8-9. doi: 10.1177/1474515115596097
37. Kramer D, Ottenberg A, Gerhardson S, et al. “Just Because We Can Doesn’t Mean We Should”: views of nurses on deactivation of pacemakers and implantable cardioverter-defibrillators. *J Interv Card Electrophysiol* 2011;32(3):243-52. doi: 10.1007/s10840-011-9596-7
38. Herman M, Horner K, Ly J, et al. Deactivation of Implantable Cardioverter-Defibrillators in Heart Failure: A Systematic Review. *Journal of Hospice & Palliative Nursing* 2018;20(1):63-71. doi: 10.1097/njh.0000000000000399
39. Javaid M, Squirrell S, Farooqi F. Improving rates of implantable cardioverter defibrillator deactivation in end-of-life care. *BMJ Open Qual* 2018;7(2):e000254.
40. Steiner JM, Bernacki G, Kirkpatrick J. My guardian angel: patients’ fears and desires related to discussing implantable cardioverter-defibrillator deactivation. *Heart* 2020;106(3):168-69. doi: 10.1136/heartjnl-2019-315935
41. Mei Ching L, Daniel PS, Joseph G, et al. Decision-Making of Patients With Implantable Cardioverter-Defibrillators at End of Life: Family Members’ Experiences. *American Journal of Hospice and Palliative Medicine®* 2016;34(6):518-23. doi: 10.1177/1049909116641622

For peer review only

For peer review only - http://bmjopen.bmj.com/site/about/guidelines.xhtml

Appendix: Example interview schedule - prospective deactivation interview

Please note the interview topic guide is a living document. The design is iterative to tailor the interview to the needs of the participant, and the issues that they feel are most important. As analysis occurs concurrently the topic guide is likely to be adapted over time - questions might be added, amended or omitted – and used flexibly.

Plan of interview

1. Introduction
2. Your experience living with an ICD
3. Reasons for deactivating the device
4. Making the decision
5. Improving conversations about deactivation
6. Feedback

1. Introduction

- Thank you agreeing to take part in this interview. As you know, we are interviewing ICD recipients who have made the decision to turn off their device. We are interested in understanding the types of information and support ICD patients need when thinking about device deactivation.
- With your permission I would like to record the interview; all details will be confidential. I am not a clinician so if there are any questions about your device it would be helpful to hear them to understand what you would like more information about but they would be best answered by your doctor or physiologist.
- There are no right or wrong answers and if you feel uncomfortable about any question we can move on to another topic or stop the interview.
- Do you have any questions or concerns?
- *Obtain written consent.*

2. Your experience living with an ICD

- Please can you tell me a little bit about your experience of having an ICD?
- What was your understanding of the device (what does it do/not do)?
- Did your experience a shock from your device at any point?
- How did your ICD impact on your quality of life?)

3. Reasons for deactivating the device

- Can you tell me a little bit about why you wanted your device turned off?
- Were there any other reasons for your decision?

4. Making the decision

- Who did you discuss turning off the device with?
- At what stage did you realise that deactivation was an option?
- What type of information did you receive?
- How was the process of deactivation explained to you?
- Had you ever thought about what would happen with your ICD as you got older? E.g. do you have an advance directive (and was the ICD incorporated into this?)
- What happened after you made the decision to have the device switched off?

For peer review only

- Did your experience match your expectations (how was it different)?

5. Improving conversations about deactivation

- From your experiences what aspects worked well?
- What aspects could have been done better?
- In your view at what stage of the pathway should the possibility of turning the device off be discussed (before/after implantation)?
- Do you have any ideas for how we might improve information giving about device deactivation?
- Who do you think should be involved in these conversations?
- If you could tell patients who are considering getting an ICD anything about what it's like, what would you tell them? What's most important for them to know in making the decision?

6. Feedback

- Thank you for your time.
- What made you take part in this interview?
- Are we asking the right questions?
- Are these the most important issues for you?
- Are there any other issues you would like to discuss to help me to better understand yours and your relative's experiences?

For peer review only

BMJ Open

'You can't start a car when there's no petrol left': A qualitative study of patient, family, and clinician perspectives on implantable cardioverter defibrillator deactivation

Journal:	*BMJ Open*
Manuscript ID	bmjopen-2020-048024.R1
Article Type:	Original research
Date Submitted by the Author:	27-Apr-2021
Complete List of Authors:	Standing, Holly ; Northumbria University, Faculty of Health and Life Sciences<br>Thomson, Richard; Newcastle University, Population Health Sciences Institute<br>Flynn, Darren; Teesside University, Centre for Rehabilitation, Exercise and Sports Science<br>Hughes, Julian; University of Bristol, Department of Population and Health Sciences<br>Joyce, Kerry; Newcastle University, Population Health Sciences Institute<br>Lobban, Trudie; Arrhythmia Alliance<br>Lord, Stephen; Newcastle Upon Tyne Hospitals NHS Foundation Trust<br>Matlock, Dan D.; University of Colorado, School of Medicine<br>McComb, Janet M; Newcastle Upon Tyne Hospitals NHS Foundation Trust<br>Paes, Paul; Northumbria Healthcare NHS Foundation Trust<br>Wilkinson, Chris; Newcastle University, Population Health Sciences Institute<br>Exley, Catherine; Newcastle University
**Primary Subject Heading**:	Cardiovascular medicine
Secondary Subject Heading:	Palliative care, Communication
Keywords:	Pacing & electrophysiology < CARDIOLOGY, Adult palliative care < PALLIATIVE CARE, Heart failure < CARDIOLOGY

SCHOLARONE™
Manuscripts

I, the Submitting Author has the right to grant and does grant on behalf of all authors of the Work (as defined in the below author licence), an exclusive licence and/or a non-exclusive licence for contributions from authors who are: i) UK Crown employees; ii) where BMJ has agreed a CC-BY licence shall apply, and/or iii) in accordance with the terms applicable for US Federal Government officers or employees acting as part of their official duties; on a worldwide, perpetual, irrevocable, royalty-free basis to BMJ Publishing Group Ltd ("BMJ") its licensees and where the relevant Journal is co-owned by BMJ to the co-owners of the Journal, to publish the Work in this journal and any other BMJ products and to exploit all rights, as set out in our licence.

The Submitting Author accepts and understands that any supply made under these terms is made by BMJ to the Submitting Author unless you are acting as an employee on behalf of your employer or a postgraduate student of an affiliated institution which is paying any applicable article publishing charge ("APC") for Open Access articles. Where the Submitting Author wishes to make the Work available on an Open Access basis (and intends to pay the relevant APC), the terms of reuse of such Open Access shall be governed by a Creative Commons licence – details of these licences and which Creative Commons licence will apply to this Work are set out in our licence referred to above.

Other than as permitted in any relevant BMJ Author's Self Archiving Policies, I confirm this Work has not been accepted for publication elsewhere, is not being considered for publication elsewhere and does not duplicate material already published. I confirm all authors consent to publication of this Work and authorise the granting of this licence.

For peer review only

'You can't start a car when there's no petrol left': A qualitative study of patient, family, and clinician perspectives on implantable cardioverter defibrillator deactivation

Holly C Standing,[a] Richard G Thomson,[b] Darren Flynn,[c] Julian Hughes,[d] Kerry Joyce,[b] Trudie Lobban,[e] Stephen Lord,[f] Daniel Matlock,[g] Janet M McComb,[f] Paul Paes,[h] Chris Wilkinson,[b] Catherine Exley[b]

Author affiliations:

[a] Department of Nursing, Midwifery and Health, Faculty of Health and Life Sciences, Northumbria University, Newcastle upon Tyne, UK

[b] Population Health Sciences Institute, Faculty of Medical Sciences, Newcastle University, Newcastle upon Tyne, UK

[c] Centre for Rehabilitation, Exercise and Sports Science, School of Health and Life Sciences, Teesside University, Tees Valley, UK

[d] Department of Population and Health Sciences, Bristol Medical School, University of Bristol, Bristol, UK

[e] Arrhythmia Alliance, Chipping Norton, Oxfordshire, UK

[f] Newcastle upon Tyne Hospitals NHS Foundation Trust, Newcastle upon Tyne, UK

[g] Department of Medicine, University of Colorado School of Medicine, Aurora, CO, USA

[h] Northumbria Healthcare NHS Foundation Trust, North Shields, UK

Corresponding author:

Professor Catherine Exley, Dean of Population Health Sciences Institute

Email: catherine.exley@ncl.ac.uk

Telephone: +44 (0) 191 2088851

Population Health Sciences Institute, Faculty of Medical Sciences, Newcastle University, Newcastle upon Tyne, NE1 7RU

For peer review only

Abstract

Objective: To explore the attitudes towards implantable cardioverter defibrillator (ICD) deactivation and initiation of deactivation discussions amongst patients, relatives and clinicians.

Design: A multi-phase qualitative study consisting of in-situ hospital ICD clinic observations, and semi-structured interviews of clinicians, patients, and relatives. Data were analysed using a constant comparative approach.

Setting: One tertiary and two district general hospitals in England.

Participants: We completed 38 observations of hospital consultations prior to ICD implantation, and 80 interviews with patients, family members, and clinicians between 2013 and 2015. Patients were recruited from pre-implantation to post-deactivation. Clinicians included cardiologists, cardiac physiologists, heart failure nurses, and palliative care professionals.

Results: Four key themes were identified from the data: the current status of deactivation discussions; patients' perceptions of deactivation; who should take responsibility for deactivation discussions and decisions; and timing of deactivation discussions. We found that although patients and doctors recognised the importance of advance care planning, including ICD deactivation at an early stage in the patient journey, this was often not reflected in practice. The most appropriate clinician to take the lead was thought to be dependent on the context, but could include any appropriately trained member of the healthcare team. It was suggested that deactivation should be raised pre-implantation and regularly reviewed. Identification of trigger points post-implantation for deactivation discussions may help ensure that these are timely and inappropriate shocks are avoided.

Conclusions: There is a need for early, ongoing, and evolving discussion between ICD recipients and clinicians regarding the eventual need for ICD deactivation. The most appropriate clinician to instigate deactivation discussions is likely to vary between patients and models of care. Reminders at key trigger points, and routine discussion of deactivation at implantation and during advance care planning could prevent distressing experiences for both the patient and their family at the end-of-life.

For peer review only

Strengths and limitations of this study (up to five)

- Implantable cardioverter defibrillator implants are increasing and existing recipients are ageing, which makes optimal end-of-life planning an important and topical issue.
- This is the first study of its kind to include both clinic observations and semi-structured interviews.
- There was a robust recruitment strategy, with a large number of participants enrolled.
- However, this study lacks the perspectives of primary care clinicians.
- A limited number of patients that were actively considering deactivation was recruited.

For peer review only - http://bmjopen.bmj.com/site/about/guidelines.xhtml

Introduction

Implantable cardioverter defibrillators (ICD) improve survival in patients at high risk of sudden cardiac death through delivering treatments to terminate life-threatening arrhythmia.[1-3] However, as people age the risk of non-arrhythmic modes of death, such as frailty and accumulating co-morbidity, progressive heart failure, or other terminal diseases such as cancer may dominate, and the potential benefit from an ICD is reduced.[4-6] More than one third of hospitalised patients with an ICD experience a ventricular tachyarrhythmia within the last hour of life,[7] and therefore leaving the device active is likely to lead to shocks at the end-of-life, which may be distressing, painful, and futile.[8-12] Advance care planning (ACP) aims to ensure that appropriate measures are in place to aid patients' transition to end-of-life care and 'a good death'.[13] Although guidelines suggest that ongoing ICD activation should be considered as part of a patient's care goals,[14 15] clinicians may be failing to engage patients sufficiently in ACP.[9 16]

A proactive approach recommended by international guidelines mandates deactivation discussion prior to implantation as a part of shared decision making,[14 17 18] This has the declared support of both clinicians and patients.[10 12 19] Yet there is evidence that this discussion does not routinely occur.[20 21] Failure to engage patients may put undue pressure on family members to make deactivation decisions, who make more than half of deactivation requests.[22] Previous work has identified that patients recall the positive, 'life-saving' attributes of ICD discussions more than pre-implant end-of-life ACP discussions.[23 24] In this study, we add to the existing evidence base by including non-participant observations in addition to semi-structured interviews. The study provides a comprehensive exploration of patient, family member and clinician attitudes towards deactivation discussions. We also explored when and how best to approach and undertake such discussions.

For peer review only - http://bmjopen.bmj.com/site/about/guidelines.xhtml

Methods

Study design

The study comprised two phases: clinical observations, and interviews with patients, family members, and clinicians. Ethical approval was granted by NHS Research Ethics Committee North East-Sunderland (Reference: 13/NE/0105). All participants provided written informed consent, and the investigation conforms with the Declaration of Helsinki.[25] Additional detail of the methods are provided in the full report.[26]

Patient and Public Involvement

Public and patient engagement was initiated prior to the development of the outline application, and their feedback guided the research question and study approach. The Founder and Trustee of Arrhythmia Alliance (TL) was a co-applicant and member of our study advisory group, and provided service user input into all stages, including co-authoring this manuscript and the report to the study funders. Two patient/carer representatives (with an interest in end-of-life issues) joined the initial project advisory group. The Arrhythmia Alliance and the North of England Cardiovascular Network reviewed the research proposal, and their comments were incorporated.

Sampling and recruitment

Phase 1: Observations

Non-participant observations [27] were conducted in clinics across three hospitals: one tertiary care (implanting) centre, and two district general hospitals. Observations at the tertiary care centre were with two implanting cardiologists, and observations at district general hospitals were with two heart failure nurses and one non-implanting cardiologist. Observations allowed researchers (HS/KJ) to familiarise themselves with the clinical environment and decision-

For peer review only

making about ICDs. Field notes informed the sampling strategy and interview schedules for phase two.

Cardiologists identified patients attending to discuss ICD implantation. Opt-out consent was used for this phase; study information was mailed with appointment letters, explaining that a researcher might be present to observe their consultation. An opt-out slip was included, and patients were asked to present this upon arrival if they chose not to participate.

Phase 2: Interviews

Clinician interviews

Clinicians were recruited from five hospitals in the north of England with interviews, conducted over the telephone or at the clinician's place of work, exploring: the referral pathway for ICD implantation; approaches to risk communication; current approaches to, and people involved in, decision-making; and timing of deactivation discussions. Purposive sampling[28] was used to capture a range of perspectives from specialities involved in the care of ICD patients.

Patient and family member interviews

We sought to include a range of patient experiences from those considering ICD implantation to those recently deactivated, as well as bereaved family members of ICD recipients 4-18 months post bereavement. The interview schedule covered issues identified through the observations and a literature review (see supplementary material), including understandings of, and feelings about, the ICD; decision-making about implantation; whether, how, and by whom deactivation had been discussed; and preferences for information and decision support.

For peer review only - http://bmjopen.bmj.com/site/about/guidelines.xhtml

Next-of-kin contact details for deceased patients with an ICD were identified and an information pack was mailed, including an introductory letter from the implanting cardiologist. The letter invited interested individuals to return a consent-to-contact form to the research team. When received, the researcher arranged an interview. Invitations offered the option of being interviewed with a friend or family member, or passing the invitation to someone more closely involved in the patient's care. Interviews were conducted at the participants' home or over the telephone and were audio-recorded, transcribed verbatim and anonymised.

Data analysis

Data collection was guided by the constant comparative method.[29] Data collection and analysis ran concurrently. Analysis of early interviews informed the interview schedule for subsequent interviews, and analysis was revised as data collection progressed. We critically examined the different perspectives and experiences of those involved in decision making about deactivation of ICDs. NVivo version 8 (QSR International, Warrington UK) was used for data management. Data were analysed by three experienced qualitative researchers (HS and KJ, with support from CE). Regular meetings were held with the rest of the research team and the advisory group to discuss themes derived from the data. All names are pseudonyms.

For peer review only

Findings

Between July 2013 and January 2015, 38 observations of consultations led by heart failure nurses, non-implanting and implanting cardiologists were conducted. Consultations lasted between 10 and 30 minutes. Forty-four patient interviews were conducted in 33 men and 11 women, aged between 47 and 85 years. Most patients (n=34) had been offered an ICD for primary prevention (patients without a history of sustained ventricular tachycardia or sudden cardiac arrest),[18] the remainder (n=10) were for secondary prevention (with a history of sustained tachycardia or sudden cardiac arrest).[18]

Table 1 provides a breakdown of patient, bereaved relative (n=7) and clinicians (n=29). Patient and bereaved relative interviews ranged from 11-113 (mean 44) minutes, clinician interviews from 20-83 (mean 42) minutes.

For peer review only

Table 1: Participant characteristics (interviews)

**Patients**	**n**
Pre-implantation	13
Declined ICD	8
Post-implantation	21
Deactivated	2
**Total**	**44**
**Bereaved relatives**	
Bereaved spouse	4
Bereaved spouse and daughter	2
Bereaved son and daughter-in-law	1
**Total**	**7**
**Clinicians**	
Implanting cardiologist	5
Non-implanting cardiologist	5
Arrhythmia nurse	1
Heart failure nurses (hospital and community)	6
Cardiac physiologists*	4
Health psychologists	2
Palliative care specialists	6
**Total**	**29**
**Overall total**	**80**

* A cardiac physiologist is a clinical scientist, known as a device (ICD or pacemaker) technician in the US.

Four key themes were identified: the current status of deactivation discussions; patients' perceptions of deactivation; responsibility for deactivation discussions and decisions; and timing of deactivation discussions.

For peer review only - http://bmjopen.bmj.com/site/about/guidelines.xhtml

Current status of deactivation discussions

Clinicians recognised the importance of timely ICD deactivation to avoid patients experiencing unnecessary shocks towards the end-of-life; however current management of ICD deactivation was considered to be sub-optimal at times. Patients were not consistently engaged in deactivation discussions and when this did occur, the patient was often close to death. Failure to deactivate the ICD in a timely manner could result in stressful and upsetting situations.

> *I think the most recent one that we've had on the ward [...] the decision was left too late, the patient was getting shocked too frequently, and nobody had had the chat to the family to say, "Well this is the defibrillator doing all of this, distressing them, and what we can do about it".* (Dr J, non-implanting cardiologist)

> *the patient in my view at that stage was actually dead, or was on the verge of being dead and it* [ICD] *was still delivering... Then once it had been deactivated, the patient passed away in a matter of a few moments. It wasn't pleasant.* (Nurse C, heart failure nurse)

Bereaved relatives felt that deactivation was often poorly handled. One suggested reason for this was clinicians' apparent reticence to deactivate the ICD prior to the patient being in the last hours of life.

> *she [cardiologist] said his heart looked alright and she didn't feel it was time to deactivate it ... She [cardiologist] said to me "Do you think that*

For peer review only - http://bmjopen.bmj.com/site/about/guidelines.xhtml

he's ready to be deactivated?" And at the time I suppose I didn't, I said no …So that wasn't a very nice experience. (Shirley Bereaved wife)

Despite having engaged in ACP with a heart failure nurse, the patient's preference for deactivation was questioned by the implanting cardiologist who deferred the decision to the patient's wife. Although the ICD was eventually deactivated, it was reported that this occurred only hours before death, following intervention by the heart failure nurse. The patient's daughter, who also participated in the interview, felt it had been inappropriate for the cardiologist to put her mother in a position where she was expected to make an immediate deactivation decision.

Some patients reported facing apparent resistance from clinicians when trying to express their preference for deactivation.

What he [the cardiologist] actually said [was], 'well, you'll basically drop down dead as soon as I switch it off'. It was so unkind. I wouldn't dare say that to anyone. (Fred, ICD deactivated)

This reported response from the cardiologist caused the patient and his wife a great deal of distress, and misrepresented the function of the ICD. The cardiologist's actions were perceived as an attempt to mislead the patient regarding the outcome of deactivation, so he would reconsider his decision to decline further medical intervention. This patient had declined his cardiologist's recommendation to be placed on the waiting list for heart transplant owing to previous experiences with organ transplantation and the associated side effects; the patient accepted he was approaching end-of-life and wanted limited intervention.

For peer review only - http://bmjopen.bmj.com/site/about/guidelines.xhtml

Rather than exploring the patient's reasons for requesting deactivation, this account would seem to demonstrate a failure to undertake SDM.

Patients' perceptions of deactivation

Understanding of, and feelings about, deactivation amongst patients and family members were variable. Some appeared very pragmatic:

> *You can't fire, start a car when there's no petrol in it, yeah? … it's no good firing your starter motor, starting your car, when there's no petrol in it.* (Janet, bereaved relative)

For these individuals, deactivation was not necessarily viewed negatively, it was a means of avoiding unpleasant and unnecessary shocks:

> *There are the end-of-life considerations* [..] *I'm aware that, that this could happen* [deactivation]. *I think, yes, if I was seriously ill and near the end-of-life. I wouldn't want the thing shocking me*. (Isobel, post-implantation)

The knowledge that deactivation may be a possibility could even offer a sense of control. However, for some patients the idea of deactivation was of concern. The device could become a source of comfort, which patients wished to keep activated as long as possible. This was particularly apparent amongst those who had recently had their ICD implanted.

> Burt: *If you were to take it off us now I'd miss it*
>
> Interviewer: *So* [you] *would never think of switching it off?*

For peer review only - http://bmjopen.bmj.com/site/about/guidelines.xhtml

Burt: *No, no, no* (Burt, post-implantation)

Appropriate timing of deactivation discussions

Pre-implantation

Pre-implantation offers the first opportunity to introduce deactivation. It was recognised that discussing deactivation could influence a patient's decision on implantation. A few patients indicated that, had they been aware of the ICD's potential to influence their mode of death and the possible need for future deactivation, this may have affected their decision making.

> *if it had been mentioned, I might have well have thought about not having one* (Adrian, post-implant)

Although most patients expressed a preference to be informed about deactivation prior to implantation, some clinicians were less convinced. They felt that discussions about deactivation and end-of-life, even when hypothetical, conflicted with perceived patient needs and expectations of doctor-patient encounters.

> *I mean most patients don't want to talk about it [EOL]… they come to see you 'cause they want to get better. They don't want to be told, "Well, you're going to die."* (Dr B, non-implanting cardiologist)

Introducing deactivation pre-implantation was viewed as an 'illogical juxtaposition', which might give mixed messages and cause distress. ICDs are offered as a potentially lifesaving intervention; to discuss end-of-life was seen as inappropriate and potentially confusing.

For peer review only - http://bmjopen.bmj.com/site/about/guidelines.xhtml

Clinicians also expressed concerns that patients might develop misconceptions that deactivation would result in immediate death.

> *I suppose also this is something to do with your heart and how people comprehend that, so it seems it can be a lot more immediate and a lot more life-threatening even at the end-of-life.* (Dr D, palliative care clinician)

Questions were also raised about patients' ability to remember information about deactivation given pre-implantation. Pre-implantation is often a time of high stress, when patients are expected to digest a considerable amount of information. Expecting patients to absorb and process information about deactivation may be unrealistic.

> *But I don't think that people at that point* [implantation] *can really take on board that at some distant point in the future that they might need it changing* (Dr E, palliative care clinician)

Ongoing and evolving discussions

Rather than one-off conversations, both patients and clinicians indicated that deactivation should be an ongoing and evolving discussion, where the appropriateness of the ICD and how it fits with the patient's life is regularly reassessed. It was thought that 'sowing the seed' of deactivation pre-implantation might facilitate later conversations.

> *you have to sow the seed, you know, plant the seed about what might happen in the future.* (Nurse C, heart failure nurse)

For peer review only - http://bmjopen.bmj.com/site/about/guidelines.xhtml

For peer review only

The point of referral for an ICD could be an initial prompt to introduce the concept of deactivation, which could be revisited at intervals. One participant suggested that 'key points in admissions' could be used as triggers for deactivation discussions. Hospital and hospice admissions, and ICD firing or replacement, could be prompts for deactivation discussions. Inclusion of the patient on palliative care registers and production of ACP documentation, such as a do not attempt cardiopulmonary resuscitation form, could be used as final trigger points where deactivation must be discussed. This approach could avoid patients 'falling through the gaps', ensuring that every patient is engaged in deactivation discussions in a timely and appropriate manner.

Responsibility for deactivation discussions and decisions

Heart failure nurses, implanting cardiologists, physiologists, palliative care clinicians, and primary care clinicians may all have a role in both advance deactivation discussions and the ultimate decision to deactivate the ICD. However, no single clinical group currently took primary responsibility for these tasks.

Although cardiologists possess a high level of expertise that may facilitate them engaging patients in these discussions, their role is traditionally focused on saving patients.

> *I think doctors don't like talking about dying, because it's a sign, it's a sign that you have failed* (Dr F, implanting cardiologist)

Further, depending on the patient's condition, contact with the cardiologist following implantation of the device may be infrequent, and so their capacity to have an ongoing role in deactivation discussions may be limited.

For peer review only - http://bmjopen.bmj.com/site/about/guidelines.xhtml

Cardiac physiologists have regular contact with ICD patients, offering an opportunity to engage patients in ongoing discussions. However, some of the physiologists interviewed appeared reticent to engage in this conversation.

> *So although I think we are in a good position in some ways, I think maybe there are other professionals out there who are in a position to do it as well.* (Mr G, cardiac physiologist)

This reticence may be related to a feeling that they lacked the requisite skills to engage patients in SDM about continuation of ICD therapy. Cardiac physiologists' confidence and comfort with regards to these responsibilities may be improved by additional training.

> *it's such a hard thing to do* [discussing deactivation and death]. *Probably need some training on how to broach the subject and when to broach it, and what to say.* (Ms I, cardiac physiologist)

Heart failure nurses felt they were already undertaking the work of engaging patients in deactivation discussions.

> *I have quite a few interesting conversations about death when I'm talking about defibrillators … it's just a simple sentence, 'your device will be switched off at some point, you can't live forever'* (Nurse H, heart failure nurse)

For peer review only - http://bmjopen.bmj.com/site/about/guidelines.xhtml

Clinicians recognised that there are various professionals who could have a role in engaging patients in deactivation discussions.

> *I think for any given patient somebody's going to be taking the lead, and it's not always clear-cut by role who that should be, so it may either be the GP* [primary care physician]*, it's conceivably a heart failure nurse, it's conceivably a MacMillan* [palliative care] *nurse or our palliative care service* (Dr M, palliative care clinician)

For peer review only - http://bmjopen.bmj.com/site/about/guidelines.xhtml

Discussion

Our findings demonstrate that clinicians understand the importance of deactivation discussions with patients and their families, and ACP for people with ICDs. Both patients and clinicians recognised the need for improvement; discussions about deactivation are often occurring late or not at all, resulting in negative impacts on patients and their families. We outline approaches for ensuring that these important and sensitive conversations take place ahead of time, based upon extensive observations and semi-structured interviews.

Previous work has shown that that patients report limited knowledge about deactivation, which is rarely discussed pre-implantation.[23 24 30 31] Yet, SDM should involve bidirectional knowledge transfer between the patient and their families, and clinicians, including elicitation of individual patient preferences and values for different outcomes.[32] Indeed, informed consent requires that patients understand the risks, benefits and consequences of interventions in the light of what is important to them in making such decisions.[33] Our findings indicate a mismatch between patients' desire to be informed about deactivation and clinicians' perception of patients' information preferences, particularly pre-implantation. Many of our patient interviewees indicated they would be comfortable discussing deactivation and believed they should be informed about this before implantation so they had appropriate expectations of the future. Knowing about the possibility of future deactivation could afford patients a sense of control. However, whilst some clinicians perceived that patients did not want early deactivation discussion, and did not routinely pursue it during consultations, our results suggest that many patients do. Furthermore, the absence of such discussion may result in patient decisional regret and contribute to late decisions about deactivation. Another potential explanation is that clinicians feel uncomfortable discussing death in a consultation

For peer review only - http://bmjopen.bmj.com/site/about/guidelines.xhtml

that is about preventing death. Knowing that many patients wish to be engaged in this discussion may help clinicians explore this pre-implantation with more confidence.

There is evidence that clinicians experience greater discomfort regarding ICD deactivation than withdrawal of other life sustaining therapies[34], and that ICDs are not considered in the same context as other end-of-life decisions.[11] It is possible that ethical questions about ICD deactivation as a form of euthanasia may contribute to some of the unease around initiating the relevant discussions.[9 35] Clinicians' reluctance to engage in end-of-life discussions may also stem from fear and anxiety of accepting the limits of their ability to save patients,[36] but perversely this may contribute to denying patients the opportunity of 'a good death'.

Our findings suggest a need for ongoing and evolving deactivation discussions, where the issue is introduced pre-implantation (as desired by the majority of patients) and built upon through subsequent encounters. Regularly revisiting ACP in relation to cardiac devices will enable clinicians to meet patients' goals of care better, recognising that these are likely to change over time with advancing years and the accumulation of health deficits.[37] However, we recognise that the increased use of remote device monitoring (in particular during the pandemic) have made this increasingly challenging. Prompts at appropriate points may help clinicians and patients to engage in these discussions,[38] and there is evidence that education, clinical tools and standardised electronic health record templates are associated with increased rates of ICD deactivation and clinicians' confidence in managing the device.[39] The possibility of empowering patients and families to initiate the discussion themselves should also be considered.

For peer review only - http://bmjopen.bmj.com/site/about/guidelines.xhtml

Our study demonstrates a lack of consensus amongst clinicians regarding responsibility for deactivation discussions. Heart failure nurses often have established relationships and regular contact with patients and their families, which may facilitate them in aligning discussions with the patient's values and preferences, and building upon the discussion over time. As others, [9 40] we suggest that a one-size-fits-all approach is not necessarily appropriate; the clinician most appropriate to discuss deactivation may differ between patients and at different times. Different models of care delivery for ICD patients will also have an influence; where specialist arrhythmia nurses are available, they will have an important role in deactivation discussions. However, these services were unavailable to the majority of our participants, resulting in more demand on the cardiologists and cardiac physiologists. Our findings support other research, which has suggested an inter-professional approach to initiating deactivation discussions.[41] Whoever is involved in care must attempt to ensure that patients do not fall through the gaps if clinicians fail to engage patients proactively in these discussions; and whichever clinicians are involved, they should have sufficient training, skills and confidence to undertake the task. Development of clear guidelines outlining the responsibility of each clinician group would provide clarity about expectations to engage in this work, and should support timely discussions within the multi-disciplinary team and between patients, clinicians, and their families. Additionally, we recognise that specialist-led secondary care clinics may lack access to comprehensive clinical information required for a holistic ongoing assessment of an individual and their circumstances. As such, we recognise the important role that general practitioners have in ACP, and the need for further data on how to optimise the interface between primary and secondary care with regard to ICD care.

For peer review only - http://bmjopen.bmj.com/site/about/guidelines.xhtml

Strengths and limitations

This article is from a large qualitative study combining observations and interviews with a range of stakeholders. The observations allowed familiarity with the clinical setting; this also allowed us to refine our sampling strategies and interview schedule for the second phase of the study (interviews). The range of participants sampled provides insight into the perspectives of various clinician groups, as well as those of patients and their family members. However, we recognise the limitations of our work. Few interviews were conducted with patients with a deactivated ICD, or considering deactivation, because there is often a short time between deactivation and death.[31] Significant attempts were made to observe clinic appointments where decisions about ICD deactivation were made, and engage these patients in interviews, but success in recruiting this group was limited. Recruitment of bereaved relatives also proved challenging. The inclusion period for this group was expanded from 4-6 months following bereavement to 4-18 months, which improved our response rate. However, some of these participants may have been less able to recall events surrounding deactivation of the ICD (or not) and death, given the length of time post-bereavement. Finally, this study did not include primary care settings; future research should explore the views and experiences of primary care clinicians regarding their role in discussions and decision-making around ICD deactivation.

For peer review only

For peer review only - http://bmjopen.bmj.com/site/about/guidelines.xhtml

Conclusions

Our study has identified significant unmet need, particularly in the area of deactivation discussion.. We believe that further work is needed in order to identify the best approach in supporting patients and clinicians to improving communication and care in this area. This is likely to include further education, development of multi-disciplinary protocols and guidelines, and reminders for both clinicians and patients to have such discussions at key points.

For peer review only

For peer review only - http://bmjopen.bmj.com/site/about/guidelines.xhtml

Contributions

Developing the study protocol: CE, JH, KJ, TL, SL, DM, JM, RT; Patient and public involvement: KJ; Developing the study design: CE, JH, KJ, TL, SL, DM, PP, RT; Data collection: HS, DF, KJ; Data interpretation: HS, CE, DF, JH, TL, SL, DM, PP, CW, RT. All authors were involved in drafting the work or revising it critically for important intellectual content. All authors approved the final version.

Competing interests

None declared.

Funding

This work was supported by The National Institute for Health Research Health Services and Delivery Research programme, 11/2004/29.

Data availability

Data are available upon reasonable request

Acknowledgements

We would like to thank all the patients, relatives, and clinicians who allowed us to observe their consultations and took part in the interviews and workshops. We are grateful to the members of the project advisory group for their valuable advice throughout the study.

For peer review only

For peer review only

For peer review only - http://bmjopen.bmj.com/site/about/guidelines.xhtml

References

1. Bardy GH, Lee KL, Mark DB, et al. Amiodarone or an implantable cardioverter-defibrillator for congestive heart failure. *N Engl J Med* 2005;352(3):225-37. doi: 10.1056/NEJMoa043399 [published Online First: 2005/01/22]
2. Kadish A, Dyer A, Daubert JP, et al. Prophylactic defibrillator implantation in patients with nonischemic dilated cardiomyopathy. *N Engl J Med* 2004;350(21):2151-8. doi: 10.1056/NEJMoa033088 [published Online First: 2004/05/21]
3. Moss AJ, Zareba W, Hall WJ, et al. Prophylactic implantation of a defibrillator in patients with myocardial infarction and reduced ejection fraction. *N Engl J Med* 2002;346(12):877-83. doi: 10.1056/NEJMoa013474 [published Online First: 2002/03/22]
4. Schleifer JW, Shen W-K. Implantable Cardioverter-Defibrillator Implantation, Continuation, and Deactivation in Elderly Patients. *Current Geriatrics Reports* 2017;6(4):279-89. doi: 10.1007/s13670-017-0226-9
5. Goonewardene M, Barra S, Heck P, et al. Cardioverter-defibrillator implantation and generator replacement in the octogenarian. *Europace : European pacing, arrhythmias, and cardiac electrophysiology : journal of the working groups on cardiac pacing, arrhythmias, and cardiac cellular electrophysiology of the European Society of Cardiology* 2015;17(3):409-16. doi: 10.1093/europace/euu248 [published Online First: 2014/10/25]
6. Walker DM, Gale CP, Lip G, et al. Editor's Choice - Frailty and the management of patients with acute cardiovascular disease: A position paper from the Acute Cardiovascular Care Association. *Eur Heart J Acute Cardiovasc Care* 2018;7(2):176-93. doi: 10.1177/2048872618758931 [published Online First: 2018/02/17]
7. Kinch Westerdahl A, Sjoblom J, Mattiasson AC, et al. Implantable cardioverter-defibrillator therapy before death: high risk for painful shocks at end of life. *Circulation* 2014;129(4):422-9. doi: 10.1161/CIRCULATIONAHA.113.002648 [published Online First: 2013/11/19]
8. Garcia M, Wanagat J. Cardiovascular Implantable Electronic Devices in End of Life Care. *Proceedings of UCLA Healthcare* 2014;18
9. Stoevelaar R, Brinkman-Stoppelenburg A, van Driel AG, et al. Implantable cardioverter defibrillator deactivation and advance care planning: a focus group study. *Heart* 2020;106(3):190-95. doi: 10.1136/heartjnl-2019-315721 [published Online First: 2019/09/21]
10. Westerdahl AK, Sutton R, Frykman V. Defibrillator patients should not be denied a peaceful death. *International journal of cardiology* 2015;182:440-46.
11. Goldstein NE, Mehta D, Teitelbaum E, et al. "It's like crossing a bridge" complexities preventing physicians from discussing deactivation of implantable defibrillators at the end of life. *J GEN INTERN MED* 2008;23 Suppl 1(1):2-6. doi: 10.1007/s11606-007-0237-x [published Online First: 2008/01/10]
12. Kelley AS, Mehta SS, Reid MC. Management of patients with ICDs at the end of life (EOL): a qualitative study. *Am J Hosp Palliat Care* 2008;25(6):440-6. doi: 10.1177/1049909108320885 [published Online First: 2008/09/25]
13. National End of Life Care Programme. Capacity, Care Planning and Advance Care Planning in Life Limiting Illness a Guide for Health and Social Care Staff. Leicester: Department of Health, Natational End of Life Care Programme, 2014.
14. Pitcher D, Soar J, Hogg K, et al. Cardiovascular implanted electronic devices in people towards the end of life, during cardiopulmonary resuscitation and after death: guidance from the Resuscitation Council (UK), British Cardiovascular Society and National Council for Palliative Care. *Heart* 2016;102 Suppl 7(Suppl 7):A1-A17. doi: 10.1136/heartjnl-2016-309721 [published Online First: 2016/06/10]
15. Beattie J. ICD deactivation at the end of life: principles and practice. A dicussion document for healthcare professionals: British Heart Foundation, 2013.

For peer review only - http://bmjopen.bmj.com/site/about/guidelines.xhtml

16. Norekval TM, Kirchhof P, Fitzsimons D. Patient-centred care of patients with ventricular arrhythmias and risk of sudden cardiac death: What do the 2015 European Society of Cardiology guidelines add? *Eur J Cardiovasc Nurs* 2017;16(7):558-64. doi: 10.1177/1474515117702558 [published Online First: 2017/04/05]
17. Padeletti L, Arnar DO, Boncinelli L, et al. EHRA Expert Consensus Statement on the management of cardiovascular implantable electronic devices in patients nearing end of life or requesting withdrawal of therapy. *Europace : European pacing, arrhythmias, and cardiac electrophysiology : journal of the working groups on cardiac pacing, arrhythmias, and cardiac cellular electrophysiology of the European Society of Cardiology* 2010;12(10):1480-9. doi: 10.1093/europace/euq275 [published Online First: 2010/08/03]
18. Al-Khatib SM SW, Ackerman MJ, Bryant WJ, Callans DJ, Curtis AB, Deal BJ, Dickfeld T, Field ME, Fonarow GC, Gillis AM, Hlatky MA, Granger CB, Hammill SC, Joglar JA, Kay GN, Matlock DD, Myerburg RJ, Page RL,. 2017 AHA/ACC/HRS Guideline for Management of Patients With Ventricular Arrhythmias and the Prevention of Sudden Cardiac Death,. *Circulation* 2017
19. Raphael CE, KOA-WING M, Stain N, et al. Implantable Cardioverter-Defibrillator Recipient Attitudes towards Device Deactivation: How Much do Patients Want to Know? *Pacing and clinical electrophysiology* 2011;34(12):1628-33.
20. Marinskis G, van Erven L, Committee ESI. Deactivation of implanted cardioverter-defibrillators at the end of life: results of the EHRA survey. *Europace : European pacing, arrhythmias, and cardiac electrophysiology : journal of the working groups on cardiac pacing, arrhythmias, and cardiac cellular electrophysiology of the European Society of Cardiology* 2010;12(8):1176-7. doi: 10.1093/europace/euq272 [published Online First: 2010/07/29]
21. Hill L, McIlfatrick S, Taylor B, et al. P2983Who decides what and when: a holistic understanding of decision-making in implantable cardioverter defibrillator deactivation. *European Heart Journal* 2017;38(suppl_1)
22. Buchhalter LC, Ottenberg AL, Webster TL, et al. Features and outcomes of patients who underwent cardiac device deactivation. *JAMA Intern Med* 2014;174(1):80-5. doi: 10.1001/jamainternmed.2013.11564 [published Online First: 2013/11/28]
23. Agard A, Lofmark R, Edvardsson N, et al. Views of patients with heart failure about their role in the decision to start implantable cardioverter defibrillator treatment: prescription rather than participation. *J Med Ethics* 2007;33(9):514-8. doi: 10.1136/jme.2006.017723 [published Online First: 2007/09/01]
24. Hill LM, McIlfatrick S, Taylor B, et al. Implantable cardioverter defibrillator (ICD) functionality: patient and family information for advanced decision-making. *BMJ Support Palliat Care* 2019:bmjspcare-2019-001835. doi: 10.1136/bmjspcare-2019-001835 [published Online First: 2019/11/28]
25. World Medical Association. World Medical Association Declaration of Helsinki: ethical principles for medical research involving human subjects. *JAMA* 2013;310(20):2191-4. doi: 10.1001/jama.2013.281053 [published Online First: 2013/10/22]
26. Standing H, Exley C, Flynn D, et al. A qualitative study of decision-making about the implantation of cardioverter defibrillators and deactivation during end-of-life care. *Health Services and Delivery Research* 2016;4(32)
27. Hammersley M, Atkinson P. Ethnography: Principles in practice: Routledge 2007.
28. Patton MQ. Qualitative Evaluation and Research Methods. Thousand Oaks: SAGE Publications 1990.
29. Charmaz K. Constructing Grounded Theory: A Practical Guide Through Qualitative Analysis. Thousand Oaks: Sage Publications 2006.
30. McEvedy SM, Cameron J, Lugg E, et al. Implantable cardioverter defibrillator knowledge and end-of-life device deactivation: A cross-sectional survey. *Palliative medicine* 2018;32(1):156-63. doi: 10.1177/0269216317718438 [published Online First: 2017/07/06]

For peer review only - http://bmjopen.bmj.com/site/about/guidelines.xhtml

31. Hill L, McIlfatrick S, Taylor B, et al. Patients' perception of implantable cardioverter defibrillator deactivation at the end of life. *Palliative medicine* 2015;29(4):310-23. doi: 10.1177/0269216314550374 [published Online First: 2014/09/23]
32. Spatz ES, Spertus JA. Shared decision making: a path toward improved patient-centered outcomes. *Circulation Cardiovascular quality and outcomes* 2012;5(6):e75-7. doi: 10.1161/CIRCOUTCOMES.112.969717 [published Online First: 2012/11/22]
33. The Supreme Court. Montgomery (Appellant) v Lanarkshire Health Board (Respondent) (Scotland). In: Court TS, ed. UKSC 11. https://www.supremecourt.uk/cases/uksc-2013-0136.html, 2015.
34. Kramer DB, Kesselheim AS, Brock DW, et al. Ethical and legal views of physicians regarding deactivation of cardiac implantable electrical devices: a quantitative assessment. *Heart rhythm* 2010;7(11):1537-42. doi: 10.1016/j.hrthm.2010.07.018 [published Online First: 2010/07/24]
35. Lebner A. Deactivating implantable cardioverter-defibrillators (ICDs) in cancer patients: A scoping review of ethical considerations. *Journal of Pain Management* 2017;10(1):99.
36. MacKenzie MA. Magical thinking around end of life: An editorial on 'Implantable Cardioverter Defibrillator deactivation discussions: Reality vs. recommendations'. *Eur J Cardiovasc Nurs* 2016;15(1):8-9. doi: 10.1177/1474515115596097 [published Online First: 2016/01/23]
37. Kramer DB, Ottenberg AL, Gerhardson S, et al. "Just Because We Can Doesn't Mean We Should": views of nurses on deactivation of pacemakers and implantable cardioverter-defibrillators. *J Interv Card Electrophysiol* 2011;32(3):243-52. doi: 10.1007/s10840-011-9596-7 [published Online First: 2011/08/02]
38. Herman M, Horner K, Ly J, et al. Deactivation of Implantable Cardioverter-Defibrillators in Heart Failure: A Systematic Review. *J Hosp Palliat Nurs* 2018;20(1):63-71. doi: 10.1097/NJH.0000000000000399 [published Online First: 2018/08/01]
39. Javaid MR, Squirrell S, Farooqi F. Improving rates of implantable cardioverter defibrillator deactivation in end-of-life care. *BMJ Open Qual* 2018;7(2):e000254. doi: 10.1136/bmjoq-2017-000254 [published Online First: 2018/05/02]
40. Steiner JM, Bernacki G, Kirkpatrick J. My guardian angel: patients' fears and desires related to discussing implantable cardioverter-defibrillator deactivation. *Heart* 2020;106(3):168-69. doi: 10.1136/heartjnl-2019-315935 [published Online First: 2019/11/02]
41. Lee MC, Sulmasy DP, Gallo J, et al. Decision-Making of Patients With Implantable Cardioverter-Defibrillators at End of Life: Family Members' Experiences. *Am J Hosp Palliat Care* 2017;34(6):518-23. doi: 10.1177/1049909116641622 [published Online First: 2016/04/02]

For peer review only

For peer review only - http://bmjopen.bmj.com/site/about/guidelines.xhtml

Appendix: Example interview schedule - prospective deactivation interview

Please note the interview topic guide is a living document. The design is iterative to tailor the interview to the needs of the participant, and the issues that they feel are most important. As analysis occurs concurrently the topic guide is likely to be adapted over time - questions might be added, amended or omitted – and used flexibly.

Plan of interview

1. Introduction
2. Your experience living with an ICD
3. Reasons for deactivating the device
4. Making the decision
5. Improving conversations about deactivation
6. Feedback

1. Introduction

- Thank you agreeing to take part in this interview. As you know, we are interviewing ICD recipients who have made the decision to turn off their device. We are interested in understanding the types of information and support ICD patients need when thinking about device deactivation.
- With your permission I would like to record the interview; all details will be confidential. I am not a clinician so if there are any questions about your device it would be helpful to hear them to understand what you would like more information about but they would be best answered by your doctor or physiologist.
- There are no right or wrong answers and if you feel uncomfortable about any question we can move on to another topic or stop the interview.
- Do you have any questions or concerns?
- *Obtain written consent.*

2. Your experience living with an ICD

- Please can you tell me a little bit about your experience of having an ICD?
- What was your understanding of the device (what does it do/not do)?
- Did your experience a shock from your device at any point?
- How did your ICD impact on your quality of life?)

3. Reasons for deactivating the device

- Can you tell me a little bit about why you wanted your device turned off?
- Were there any other reasons for your decision?

4. Making the decision

- Who did you discuss turning off the device with?
- At what stage did you realise that deactivation was an option?
- What type of information did you receive?
- How was the process of deactivation explained to you?
- Had you ever thought about what would happen with your ICD as you got older? E.g. do you have an advance directive (and was the ICD incorporated into this?)
- What happened after you made the decision to have the device switched off?

For peer review only

- Did your experience match your expectations (how was it different)?

5. Improving conversations about deactivation

- From your experiences what aspects worked well?
- What aspects could have been done better?
- In your view at what stage of the pathway should the possibility of turning the device off be discussed (before/after implantation)?
- Do you have any ideas for how we might improve information giving about device deactivation?
- Who do you think should be involved in these conversations?
- If you could tell patients who are considering getting an ICD anything about what it's like, what would you tell them? What's most important for them to know in making the decision?

6. Feedback

- Thank you for your time.
- What made you take part in this interview?
- Are we asking the right questions?
- Are these the most important issues for you?
- Are there any other issues you would like to discuss to help me to better understand yours and your relative's experiences?

For peer review only
